# Faster Smith-Waterman database searches with inter-sequence SIMD parallelisation

**DOI:** 10.1186/1471-2105-12-221

**Published:** 2011-06-01

**Authors:** Torbjørn Rognes

**Affiliations:** 1 Department of Informatics, University of Oslo, PO Box 1080 Blindern, NO-0316 Oslo, Norway; 2 Centre for Molecular Biology and Neuroscience (CMBN), Department of Microbiology, Rikshospitalet, Oslo University Hospital, PO Box 4950 Nydalen, NO-0424 Oslo, Norway; 3 Sencel Bioinformatics AS, PO Box 180 Vinderen, NO-0319 Oslo, Norway

## Abstract

**Background:**

The Smith-Waterman algorithm for local sequence alignment is more sensitive than heuristic methods for database searching, but also more time-consuming. The fastest approach to parallelisation with SIMD technology has previously been described by Farrar in 2007. The aim of this study was to explore whether further speed could be gained by other approaches to parallelisation.

**Results:**

A faster approach and implementation is described and benchmarked. In the new tool SWIPE, residues from sixteen different database sequences are compared in parallel to one query residue. Using a 375 residue query sequence a speed of 106 billion cell updates per second (GCUPS) was achieved on a dual Intel Xeon X5650 six-core processor system, which is over six times more rapid than software based on Farrar's 'striped' approach. SWIPE was about 2.5 times faster when the programs used only a single thread. For shorter queries, the increase in speed was larger. SWIPE was about twice as fast as BLAST when using the BLOSUM50 score matrix, while BLAST was about twice as fast as SWIPE for the BLOSUM62 matrix. The software is designed for 64 bit Linux on processors with SSSE3. Source code is available from http://dna.uio.no/swipe/ under the GNU Affero General Public License.

**Conclusions:**

Efficient parallelisation using SIMD on standard hardware makes it possible to run Smith-Waterman database searches more than six times faster than before. The approach described here could significantly widen the potential application of Smith-Waterman searches. Other applications that require optimal local alignment scores could also benefit from improved performance.

## Background

The alignment of two biological sequences is a fundamental operation that forms part of many bioinformatics applications, including sequence database searching, multiple sequence alignment, genome assembly, and short read mapping.

Smith and Waterman [1] described a simple and general algorithm requiring O(*N*^3^) time and O(*N*^2^) memory to identify the optimal local sequence alignment score using a substitution score matrix and a general gap penalty function. Gotoh [2] showed that with affine gap penalties the optimal local alignment score could be computed in just O(*N*^2^) time and O(*N*) memory.

When the optimal alignment score needs to be computed many times, for example when searching a sequence database, the computation time becomes substantial. Several approaches have been pursued to reduce the time needed. Heuristic approaches like BLAST [3,4] are considerably faster, but are not guaranteed to discover the optimal alignment.

Reconfigurable hardware in the form of FPGA (Field-Programmable Gate Array) can also accelerate the speed of alignment score computations. Li *et al*[5]. reported speeds equivalent to about 23.8 billion cell updates per second (GCUPS) with DNA sequences, on a state-of-the-art FPGA board. Search speed is often reported in GCUPS, which indicates the billion (giga) number of cells in the alignment matrix (query sequence length times total number of database residues), processed per second.

The algorithm can also be implemented with various forms of parallelisation in software running on more common hardware. Pairwise alignment of separate sequences is in principle "embarrassingly" parallel because the computations for each pair of sequence are completely independent. Alpern *et al*[6]. suggested improving speed by performing several independent alignment score computations in parallel by dividing the bits of wide registers into several narrower units and using instructions to perform arithmetic operations on these units individually. This form of parallelism within a register was later made much simpler and easier by microprocessor manufacturers with the introduction of technologies like MMX, SSE, SSE2, MAX, MVI, VIS, and AltiVec, which are now generally referred to as SIMD technology. Several implementations take advantage of the SSE2 instructions available on Intel processors [7]. The approach where parallelisation is carried out across multiple database sequences is also known as inter-task parallelisation, in contrast to intra-task parallelisation where the parallelisation occurs within a single pair of sequences.

**Figure 1:**
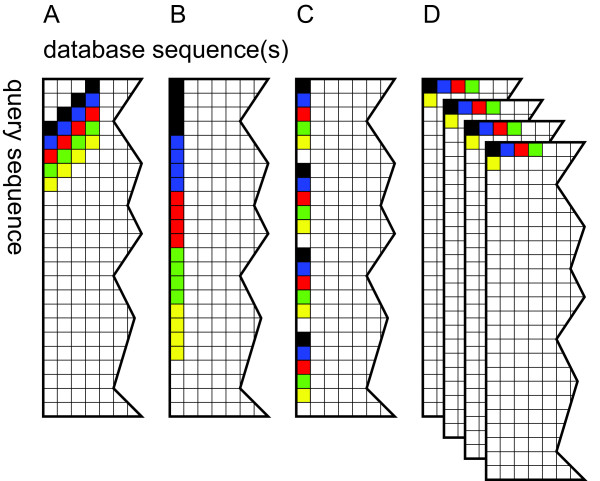
**Approaches to vectorisation of Smith-Waterman alignments**. Alignment matrices are shown with the elements that form the first five vectors processed indicated in black, blue, red, green and yellow. For simplicity, vectors of only 4 elements are shown, while 16 elements would normally be used. (A) Vectors along the anti-diagonal, described by Wozniak *et al*[8]. (B) Vectors along the query, described by Rognes and Seeberg [9]. (C) Striped approach, described by Farrar [10]. (D) Multi-sequence vectors, described by Alpern *et al*[6]. and in this paper.

Efforts have since mostly concentrated on parallelisation within a single alignment of one pair of sequences. Figure 1 illustrates the main approaches. Wozniak [8] suggested computing cells along the minor diagonal in the alignment matrix in parallel because these calculations are independent. Rognes and Seeberg [9] found that using cells along the query sequence was faster despite some data dependences, because loading values along the minor diagonal was too complicated. Farrar [10] introduced a "striped" approach where computations were carried out in parallel in several separate stripes covering different parts of the query sequence to reduce the impact of some of the computational dependencies. Farrar's striped approach is generally the fastest, and he has reported speeds of more than 11 and 20 GCUPS on four and eight cores, respectively [11]. Szalkowski *et al*[12]. described the SWPS3 implementation which uses the striped approach of Farrar, claiming speeds of up to 15.7 GCUPS on a quad-core processor. The performance was highly dependent on query length though. Using the P07327 query sequence, with a typical protein length of 375 residues, SWPS3 performance was roughly 9 GCUPS.

The Cell processor manufactured by Sony, Toshiba and IBM, have one main core (Power Processing Element, PPE) and 8 minor cores (Synergistic Processing Elements, SPEs). These cores have SIMD vector processing capabilities. Cell processors are found in some IBM servers (QS20) as well as the Sony PlayStation 3 (PS3) (only 6 SPEs available). Several implementations for this processor have been described. SWPS3 by Szalkowski *et al*[12]. used the Farrar approach and claimed speeds on the PS3 of up to 8 GCUPS. Also here the performance was highly dependent on query length. With the P07327 query sequence, SWPS3 performance was less than 2 GCUPS on the PS3. Wirawan *et al*[13]. also employed the Farrar approach in their implementation called CBESW and claimed speeds on the PS3 of over 3.6 GCUPS, while the performance was 2.2 GCUPS with the P07327 query sequence. Farrar also reported speeds of 15.5 GCUPS on an IBM QS20 and up to 11.6 GCUPS on the PS3 using the same query [11]. Rudnicki *et al*[14]. described an implementation that used parallelisation over multiple database sequences on the PS3 and reported speeds approaching 9 GCUPS.

Graphics processors (GPUs) can also accelerate alignments. Several implementations have employed the CUDA interface to Nvidia GPUs. The CUDASW++ tool by Liu *et al*[15]. reportedly performed 9.5 GCUPS on the single-GPU GeForce GTX 280 and 14.5 GCUPS on the dual-GPU GeForce GTX 295. Ligowski and Rudnicki [16] reported speeds of up to 14.5 GCUPS on the dual-GPU GeForce 9800 GX2. In 2010, Liu *et al*[17]. reported speeds up to 17 and 30 GCUPS by CUDASW++ 2.0 on the single-GPU GeForce GTX 280 and the dual-GPU GeForce GTX 295, respectively. Recently, Ligowski *et al*[18]. reported a speed of 42.6 GCUPS on the GeForce GTX 480.

The main advantage of the inter-task parallelisation approach where multiple database sequences are processed in parallel as described by Alpern *et al*[6]. is that it simply avoids all data dependences within the alignment matrix. This approach does not seem to have been explored much in later implementations using SIMD technology apart from the work by Rudnicki *et al*[14]. However, in the GPU-based tools [15,16] this approach is common. The aim of the present study was to explore the use of this approach further using SIMD on ordinary CPUs.

Here the algorithm is implemented on Intel processors with SSSE3 [7] with parallelisation over multiple database sequences as illustrated in Figure 1D. Instead of aligning one database sequence against the query sequence at a time, residues from multiple database sequences are retrieved and processed in parallel. Rapid extraction and organisation of data from the database sequences have made this approach feasible. The approach has been implemented in a tool called SWIPE. The approach also involves computing four consecutive cells along the database sequences before proceeding to the next query residue in order to reduce the number of memory accesses needed.

The performance of the new implementation has been extensively tested using different scoring matrices, gap penalties, query sequences and number of threads. The speed of SWIPE was almost constant at more than 100 GCUPS on a dual Intel Xeon X5650 six-core processor system for a wide range of query lengths. SWIPE was about six times faster than SWPS3 and Farrar's own implementation for a typical length query, but the factor varied between 2 and 18 depending on query length and number of threads used. Two versions of BLAST were tested and the speed was found to be highly dependent on the score matrix. SWIPE was about twice as fast as BLAST using the BLOSUM50 matrix, while BLAST was about twice as fast as SWIPE using the BLOSUM62 matrix.

## Methods

### Benchmarking

Benchmarking was performed on compute nodes in the Titan high performance cluster at the University of Oslo. Entire nodes were reserved to ensure that no other major processes were running. All data was initially copied to a fast local disk to reduce the influence of the computer networks and minimize file reading time. The output from all programs was redirected to/dev/null to minimize performance differences due to the amount of output. All combinations of programs, number of threads, query sequences, score matrices, gap open penalties and gap extension penalties were run 15 times and the median total wall clock execution time was recorded.

### Software

Table 1 lists the software packages that were benchmarked, including their version numbers and command line options used.

**Table 1:** Programs included in performance testing

Program	Version	Command line
BLAST	2.2.24	./blastall -p blastp -F F -C 0 -b 0 -v 10 -a $T -M $M -G $GO -E $GE -i $Q -d $D
BLAST+	2.2.24+	./blastp -seg no -comp_based_stats F -num_alignments 0 -num_descriptions 10 -num_threads $T -matrix $M -gapopen $GO -gapextend $GE -query $Q -db $D
SWIPE	1.0	./swipe -v 10 -a $T -M $M.mat -G $GO -E $GE -i $Q -d $D
STRIPED		./striped -c 10 -T $T -i -$GO -e -$GE $M.mat $Q $D.fsa
SWPS3	20080605	./swps3 -j $T -i -$GO -e -$GE $M.mat $Q $D.fsa

Command line variables: threads ($T), score matrix file name ($M), gap open ($GO) and extension ($GE) penalties (positive values), query file name ($Q), database file basename ($D)

SWIPE was written mainly in C++ with some parts hand coded in inline assembler and some using SSE2 and SSSE3 intrinsics. It was compiled for 64 bit Linux using the Intel C++ compiler version 11.1. Source code is available at http://dna.uio.no/swipe/ under the GNU Affero General Public License, version 3. An executable binary and score matrix files are also available at the same location. The same files are included in a gzipped tar archive as additional file 1.

The source code for Farrar's STRIPED software was downloaded from the author's website and compiled with the GNU gcc compiler as specified in the supplied Makefile [11]. The Makefile was also modified to compile STRIPED using the Intel compiler, to see if there were any differences in performance.

The binary executable for the SWPS3 program was downloaded from the authors' website and used directly [12].

Precompiled binaries for BLAST and BLAST+ were downloaded from the NCBI FTP site [4].

Graphs were drawn using Gnuplot version 4.5.

### Hardware

Performance tests were carried out on Dell PowerEdge M610 blade servers with 48 GB RAM and dual Intel Xeon X5650 six-core processors running at 2.67 GHz. The X5650 processors have simultaneous multithreading capability also known as hyper-threading (HT). With HT enabled, each of these computers has a total of 24 logical cores.

### Threads

The programs were run using 1 to 24 threads. To take full advantage of the hardware a number of threads equal to the number of logical cores is usually the most appropriate. There are however important differences between software on the effect of HT and in the ability to make efficient use of the cores available. To simplify comparisons, some of the tests were only performed with 1 or 24 threads.

### Database sequences

UniProt Knowledgebase Release 11.0 [19] consisting of both Swiss-Prot release 53.0 and TrEMBL release 36.0 of 29 May 2007 was used for the performance tests. This database consists of 4 646 608 protein sequences with a total of 1 517 383 530 amino acid residues. The longest sequence contains 36 805 residues.

This database was chosen because the size was large enough to be realistic but smaller than the apparent 2 GB file size limit of some software. The database also did not contain any of the special J, O or U amino acid residue symbols that some of the software could not handle. Finally, this release of the database should be available for download in the foreseeable future, making it suitable also for future benchmarking.

The current version of SWIPE will not work with databases split by formatdb into separate volumes. SWIPE therefore cannot search databases larger than about 4 billion amino acids.

The database was converted into FASTA format by a simple Perl script and then formatted with NCBI formatdb version 2.2.24 into the NCBI BLAST binary database format [4]. This binary format was read by SWIPE, BLAST and BLAST+, while STRIPED and SWPS3 read the FASTA-formatted database file directly.

### Query sequences

The 32 query sequences with accession numbers P56980, O29181, P03630, P02232, P01111, P05013, P14942, P00762, P53765, Q8ZGB4, P03989 (replacing the identical but obsolete P10318), P07327, P01008, P10635, P58229, P25705, P03435, P42357, P21177, Q38941, O60341, P27895, P07756, P04775, P19096, P28167, P0C6B8, P20930, P08519, Q7TMA5, P33450 and Q9UKN1, ranging in length from 24 to 5478 residues were retrieved from the UniProt database [18]. Most of them have previously been used several times for performance testing. To simplify comparisons, some of the tests were only performed with the 375 residues long P07327 query, representing a protein of roughly average length.

### Score matrices and gap penalties

All 82 different combinations of amino acid substitution score matrices and gap penalties accepted by BLAST were tested. The matrices used were BLOSUM45, BLOSUM50, BLOSUM62, BLOSUM80, and BLOSUM90 from the BLOSUM series [20] as well as PAM30, PAM70, and PAM250 from the PAM series [21]. Matrices were obtained from the NCBI FTP site. Rows and columns for stop codons (*) were removed from the matrices for compatibility with the SWPS3 program. SWPS3 would only run successfully using the BLOSUM45, 50 and 62 matrices. To simplify comparisons, some of the tests were only performed with the BLOSUM62 matrix and gap open and extension penalties of 11 and 1, respectively, which is the BLAST default.

## Results

### Algorithm

The optimal local alignment score of two sequences can be computed using a dynamic programming approach. The recurrence relations for the algorithm of Smith and Waterman [1] with the modifications of Gotoh [2] for affine gap penalty functions are shown below.(1)(2)(3)(4)

The query sequence *q *of length *m *contains residues *q_i _*. The database sequence *d *of length *n *contains residues *d_j _*. *H_i,j _*is the score for aligning the prefixes of *q *and *d *ending in the alignment of residues *q_i _*and *d_j _*. *E_i,j _*and *F_i,j _*are the scores of aligning the same prefixes of *q *and *d *but ending with a gap in the query and database sequence, respectively. *P*[*q_i , _d_j _*] is the score of aligning residues *q_i _*and *d_j _*with each other according to the substitution score matrix *P*. *Q *is the sum of gap open and extension penalties while *R *is the gap extension gap penalty. *S *is the overall optimal local alignment score.

The calculations are carried out column by column. Only parts of the *H*, *E *and *F *matrices need to be kept in memory: a single element of the *F *matrix as well as two arrays containing *m *elements each, corresponding to one column of the *H *and *E *matrices.

### Implementation

The main features of the implementation are described below.

#### Parallelisation over sixteen database sequences

**Figure 2:**
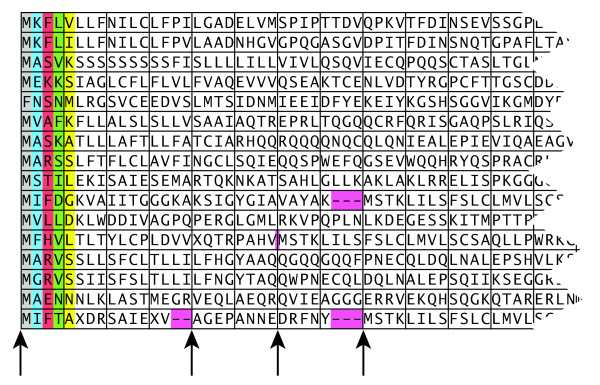
**Blocks of database residues processed together**. The residues of the first five vectors processed are indicated on grey, blue, red, green and yellow background. Four symbols from each of the database sequences form blocks that are processed as a group. Padding of some sequence blocks are indicated with dashes on a pink background. An additional database sequence change is indicated with a pink vertical bar. Arrows below the sequences indicate positions were new sequences begin.

Residues from 16 different database sequences are processed in parallel as indicated in Figure 1D. These 16 residues are all simultaneously compared to the same query residue. The operations are carried out using vectors consisting of 16 independent bytes. The 16 residues are fed into sixteen independent channels. When the first of these sixteen database sequences ends, the first residue of the next database sequence is loaded into the channel. The databases sequences are read in the order they are found in the original database file. In contrast to the approach by Rudnicki *et al*. [14], the database is not sorted by sequence length. Figure 2 illustrates this approach.

#### Compact core code of ten instructions

**Figure 3:**
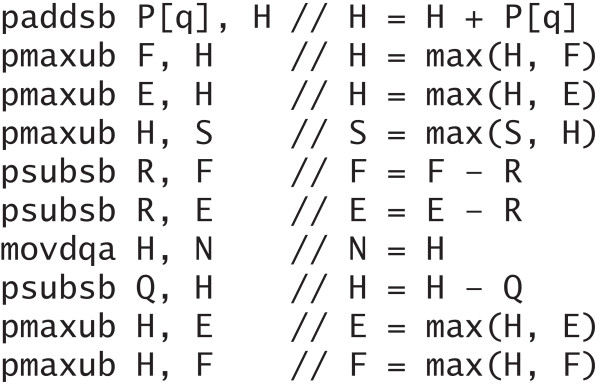
**Core instructions**. These are the ten core instructions executed for each vector of cells in the alignment matrices. In this code, the first (left) operand is the source and the second (right) operand is the destination.

The basis for the computations of the values in each cell in the alignment matrices are the recurrence relations described in the Algorithm section. The computations can be written in as little as ten assembly instructions that constitute the core of the inner loop of the computations, as shown in Figure 3. These ten instructions compute in parallel the values for each vector of 16 cells in independent alignment matrices. The exact selection of instructions and their order is important; this part of the code was therefore hand coded in assembler to maximise performance. In the figure, *H *represents the main score vector. The *H *vector is saved in the *N *vector for the next cell on the diagonal. *E *and *F *represent the score vectors for alignments ending in a gap in the query and database sequence, respectively. *P *is the vector of substitution scores for the database sequences versus the query residue *q *(see temporary score profiles below). *Q *represents the vector of gap open plus gap extension penalty. *R *represents the gap extension penalty vector. *S *represents the current best score vector. All vectors, except *N *are initialised prior to this code.

#### Processing four consecutive cells along the database sequences

**Figure 4:**
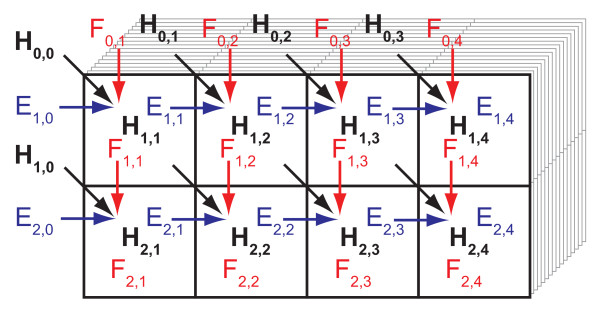
**Block of cells computed in each iteration**. In each iteration of the inner loop blocks of eight cells in the alignment matrices computed. The *H*, *E*, *F *and *S *values are updated for each cell. The computations start in the upper left cell, proceed right three times and then continue from the left on the second row.

During the computations of the matrix cells, the values in the two arrays with the *H *and *E *values usually have to be read and written once for each matrix cell. These arrays are usually small enough to be cached at a close cache level, so the memory access time should not be a major concern, but they still need to be written and read back for each cell. Since there are sixteen 128-bit xxm registers available and ample space for keeping the *H*, *E *and *F *values of a few cells in the registers, it is possible to reduce running time somewhat by computing a few consecutive cells along the database sequences before moving on to the next query residue. Four consecutive cells was found to perform well. Unrolling the inner loop once along the query sequence was also found to work well. The basic computing blocks then consist of two times four cells that are processed in each inner loop iteration as shown in Figure 4.

#### Updating scores and padding blocks

When a new database sequence begins in one of the channels, the score of the previous database sequence must be recorded. In addition, the *H*, *E *and *S *scores from the previous sequence must be reset. When a new column is going to be processed, it is checked whether any database sequence ended in the previous column. If that is the case, special processing is carried out. The score of the sequences that ended are recorded. A mask is created and later used to reset the values of *H*, *E *and *S *in the appropriate channels before the new column is processed. The channels are filled, and one or more new sequences are started. A somewhat simpler and faster processing step is carried out for the new column if no sequences ended in the previous column. In the simpler processing step no scores need to be saved, no new sequences are started, and no mask need to be created or used. Since most columns are of the simpler type the overall performance is mostly dependent on the speed of processing the simple columns. To simplify computations, each channel is padded with 1-3 null symbols after the end of a sequence if its length is not a multiple of 4, to ensure that a new database sequence will only begin at the beginning of a new block of cells. This padding is indicated in pink in Figure 2. The padding increases the total number of cell a little bit, but allows the checks described above to be carried out only on every fourth residue.

#### Computation of temporary score profiles

**Figure 5:**
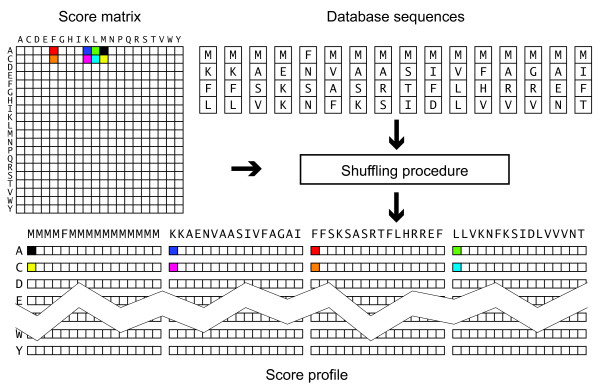
**Creation of a temporary score profile for 64 database sequence residues**. A standard score matrix and 4 residues from 16 different database sequences form the basis for a kind of temporary partial database sequences score profile. The score profile enables rapid comparison of any query residue with these 64 database residues.

To make computations fast, it is essential that the vectors of substitution score values can be loaded quickly. Each score vector corresponds to the score of a single query residue against 16 residues from different database sequences. A kind of temporary score profile is created as outlined in Figure 5. This score profile is valid for matching any query residue with 4 successive residues from 16 database sequences. For every fourth residue in the database sequences a new score profile must be constructed.

The temporary score profiles are created from the ordinary substitution score matrices (e.g. BLOSUM62) and the 4 × 16 database sequence residues using a series of packed shuffle instructions (pshufb). The shuffle instruction is only available on Intel processors with Supplemental Streaming SIMD Extensions 3 (SSSE3). On processors without SSSE3 (i.e. AMD processors and older Intel processors), the computations may be replaced by a kind of matrix transpose operation using a series of unpack instructions (punpcklbw, punpckhbw) with only a modest speed penalty.

#### Score range and selection of arithmetic instructions

Computations are initially performed using only a 7 bit score range. This allows 16 alignment score matrices to be computed in parallel using SSE2 instructions. Additions and subtractions are performed using signed and saturated arithmetics, while the maximum operations are carried out on unsigned numbers. Only the 7 bit score range from -128 to -1 (signed numbers) or 128 to 255 (unsigned numbers) is used. All scores are biased by an offset of 128. This range of values will ensure that signed saturated addition and subtraction works well on the lower boundary. Also, the unsigned maximum works well in this range. The range enables the use of the packed maximum unsigned byte instruction (pmaxub) and packed add and subtract signed saturated bytes instructions (paddsb and psubsb), which are available on all SSE2 processors and gives the highest speed.

An 8 bit range, which would allow the same number (16) of parallel computations as a 7-bit range, and at the same time allow a wider score range, is not used because it is slower. Either the range from -128 to 127, or the range from 0 to 255 could be used. There are both signed and unsigned versions of the instructions for parallel computation of the maximum of bytes (pmaxub, pmaxsb) and for parallel addition and subtraction of bytes (paddusb, psubusb, paddsb, psubsb), but the pmaxsb instruction for the maximum of signed bytes was only recently available in SSE4.1 and is slower than pmaxub. Unsigned additions and subtractions (paddusb and psubusb) may be used, but the addition of the score matrix vector then requires two instructions. First the score vector including a bias must be added; then the bias must be subtracted.

Versions using 16-bit and 63-bit score ranges are also implemented and used when overflow is detected in computations with lower score ranges. The 16-bit version allows 8 parallel computations. When potential overflow is detected in computations with a narrow score range, the alignment score for that database sequence is recalculated using the next wider score range (first 16-bit and then 63-bit if necessary). Because rather few sequences will usually reach a score that cannot be represented by 7 bits, the additional computation time for wider score ranges are usually negligible.

Recalculations with a wider score range are carried out on a subset of sequences after all sequences in a chunk of database sequences (see below) have been processed using the narrower score range.

#### Reading the sequence database

Database sequences were stored in the NCBI BLAST database format, produced by the formatdb tool. This is a binary format where the sequence information is split into at least 3 files: indices (.pin), headers (.phr) and sequences (.psq). The file format allows efficient reading of sequences into memory. Protein sequences are stored using byte values in the range 1-24 and 26-27 representing amino acid residues A-I, K-N, P-T, V-Z, U, O and J, respectively. Sequences are separated by a zero byte, which simplifies the check for sequence ends.

Database sequences are retrieved using memory mapping of the .pin and .psq files. This is an efficient and convenient method of accessing the sequences, in which the operating system manages reading data into memory from disk as necessary, concurrently with program execution. The sequence database is divided into 100 chunks per thread. Each chunk contains approximately the same number of sequences. The sequences in one chunk are mapped into memory and processed before the next chunk is mapped. This results in a small memory footprint of the program.

#### Multiple threads

SWIPE uses multiple threads (pthreads) that work on different parts of the sequence database. The number of threads is specified when starting the program and should in general be equal to the number of cores of the computer. For the latest generations of Intel processors with hyper-threading, a number of threads equal to the number of logical cores is usually most effective. Chunks of database sequences are assigned to the threads as they are ready for more work, so the threads may not process exactly the same number of chunks each. Results from the threads are inserted into a common hit list after each chunk is processed.

### Testing

The SWIPE software was benchmarked against BLAST, BLAST+, STRIPED and SWPS3 under many different conditions to measure speed. The performance using a variable number of threads and the effect of query sequence length was studied. Additionally, the impact of different scoring systems, both substitution score matrices and gap open and extension penalties was examined.

#### Threads

**Figure 6:**
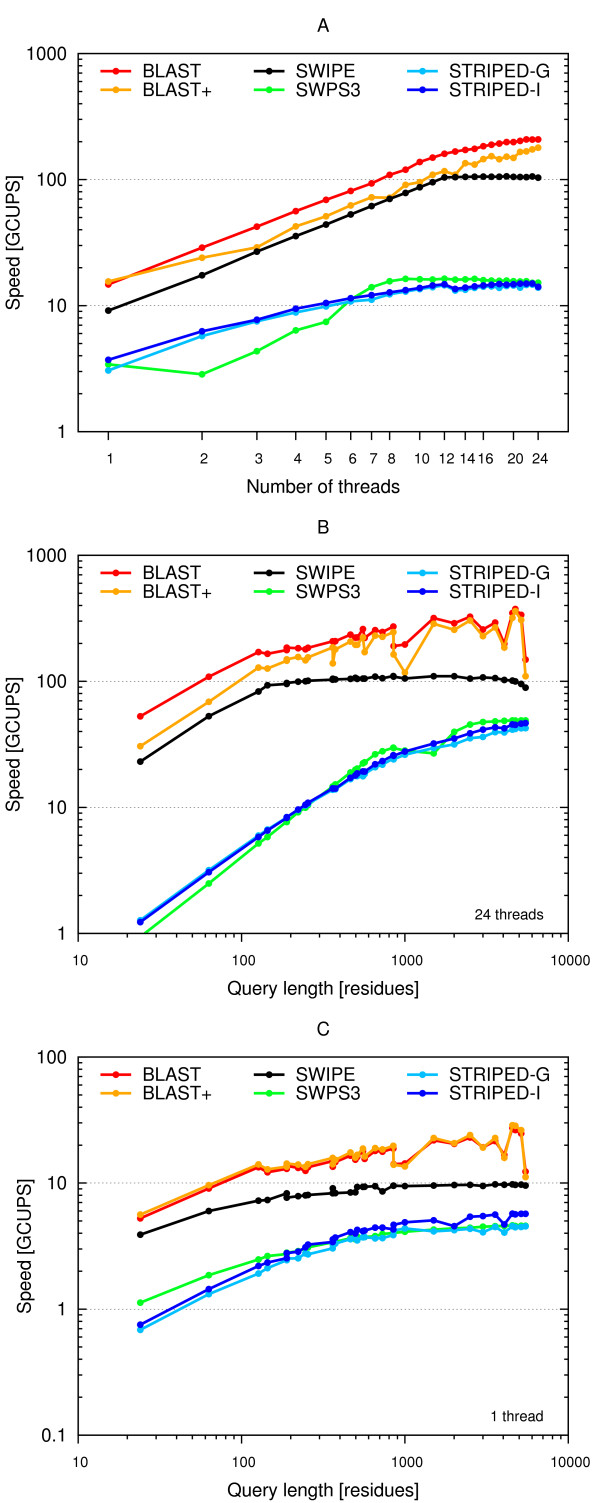
**Performance dependency on number of threads and query length**. The speed in billion cell updates per second (GCUPS) of the BLAST (red), BLAST+ (orange), SWIPE (black), and SWPS3 (green) programs, as well as STRIPED compiled with the GNU (light blue) and Intel (dark blue) compiler, using a variable number of threads and queries of varying length. (A) Number of threads ranging from 1 to 24 and the 375 residue long P07327 query sequence. (B) Query sequences ranging from 24 to 5478 residues in length and 24 threads. (C) Query sequences of varying length and 1 thread. All scales are logarithmic. The BLOSUM62 matrix and gap open and extension penalties of 11 and 1, respectively, were used in all cases.

Figure 6A indicates the performance of the programs running with 1 to 24 threads, the 375 residue long P07327 query sequence, the BLOSUM62 matrix, and with gap open and extension penalties of 11 and 1, respectively. SWIPE runs at 9.1 GCUPS using a single thread and reaches its maximum performance with 19 threads at 106.2 GCUPS, but there is almost no gain from additional threads beyond 12. It scales very well and the maximum speed-up (the ratio of maximum speed to single thread speed) is 11.6. SWPS3 runs at 3.4 GCUPS using a single thread and reaches its best performance at 12 threads with 16.4 GCUPS, but has little gain beyond 9 threads. The maximum speed-up is 4.8. Surprisingly, the speed using two threads is inferior to that with a single thread. STRIPED compiled with the GNU compiler runs at 3.1 GCUPS with a single thread and reaches its maximum performance with 23 threads at 14.7 GCUPS, but gains little beyond 12 threads. The maximum speed-up is 4.8. Compiling STRIPED with the Intel compiler resulted in a 21% speed increase to 3.7 GCUPS when running on a single thread, but just 2% to 15.0 GCUPS with 23 threads. This corresponds to a maximum speed-up of 4.0. BLAST and BLAST+ run at 14.7 and 15.5 GCUPS, with a single thread, respectively, scale very well and reach their maximum performance when 24 threads are running with speeds of 208.4 and 178.9 GCUPS, respectively. The maximum speed-ups of BLAST and BLAST+ are 14.2 and 11.5, respectively.

#### Query length

Figure 6B and Figure 6C illustrates the performance with queries of varying length using either 24 threads (B) or a single thread (C). 32 different sequences with lengths ranging from 24 to 5478 amino acid residues were used as queries.

SWIPE had a rather flat performance curve. For queries shorter than about 100 residues there was a gradual loss in performance, especially when running with many threads. The speed was also slightly reduced for very long queries when using many threads. The performance ranged from 23.1 to 110.1 GCUPS for 24 threads and from 3.9 to 9.8 GCUPS for a single thread.

The SWPS3 program was very dependent on query length with speeds ranging from 0.94 to 49.0 GCUPS on 24 threads and between 1.1 and 4.6 GCUPS on a single thread.

The STRIPED program compiled with the Intel compiler was also quite dependent on the query length, in particular when running on 24 threads, with speeds ranging from 1.2 to 46.6 GCUPS. On a single thread, the speed of STRIPED varied between 0.8 and 5.7 GCUPS. STRIPED compiled with the GNU compiler was in general slightly slower, particularly with longer queries.

The speed of the BLAST programs seemed somewhat faster with longer query sequences with speeds ranging from 52.8 to 374.1 and 30.6 to 360.2 GCUPS for BLAST and BLAST+, respectively, but the performance varied a bit from sequence to sequence. There was a noticeable drop in performance with queries shorter than about 100 residues.

#### Scoring systems

**Figure 7:**
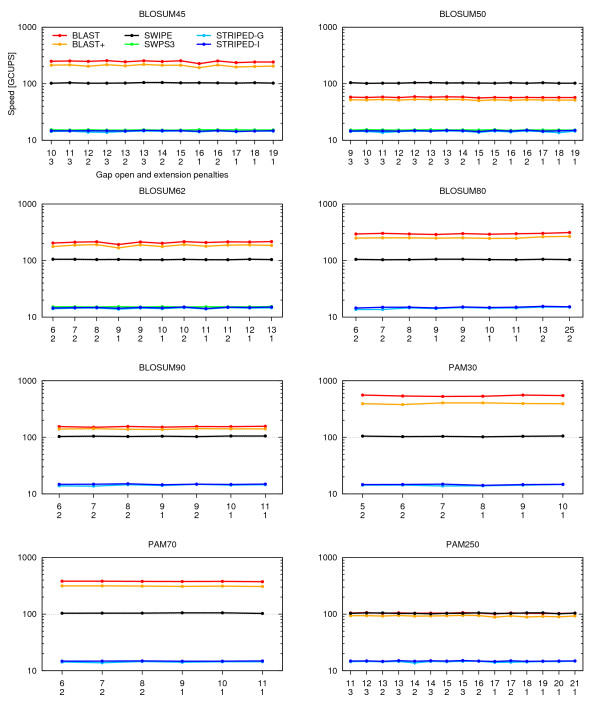
**Performance with different scoring systems**. The speed in billion cell updates per second (GCUPS) (logarithmic scale) is shown for the BLAST (red), BLAST+ (orange), SWIPE (black), and SWPS3 (green) programs, as well as STRIPED compiled with the GNU (light blue) and Intel (dark blue) compiler, using different scoring systems. All combinations of scoring matrices and gap penalties accepted by BLAST were tested. The matrix name is indicated above each graph, while the open and extension gap penalties are indicated on the x-axis. The query sequence was P07327 and 24 threads were running. SWPS3 would not run successfully in all cases.

Figure 7 shows the performance under different scoring systems. All combinations of matrices and gap penalties allowed by BLAST were tested. The 375 residue long P07327 query sequence and 24 threads were used. The performance of SWIPE is almost constant at about 102-106 GCUPS. The performances of STRIPED and SWPS3 are also almost constant at about 14-15 and 15 GCUPS, respectively. The performance of BLAST was highly dependent on the scoring matrix used. SWIPE was almost twice as fast as ordinary BLAST using the BLOSUM50 matrix. The speeds of the two programs were quite similar with the PAM250 matrix, while BLAST was faster for the other matrices. In general, BLAST+ was about 10-20% slower than ordinary BLAST. Gap penalties had little impact on performance in general, but relatively low gap penalties seemed to reduce the speed of BLAST, BLAST+, and STRIPED in a few cases (e.g. BLOSUM62 with gap penalties 9 and 1), while the impact on SWIPE and SWPS3 was negligible.

## Discussion

The SWIPE software greatly increases the speed of sequence database searches based on the Smith-Waterman algorithm compared to earlier SIMD implementations, being more than six times faster in realistic cases. SWIPE was found to be performing at a speed of 106 GCUPS with a 375 residue query sequence on a dual Intel Xeon X5650 six-core processor system. The speed was a bit dependent on the query length, but independent of the scoring system used. The maximum speed corresponds to the processing of more than 3.3 cells per physical core in each clock cycle.

Using GPUs, speeds of up to 42.6 GCUPS have been reported for CUDASW++ 2.0 on a Nvidia GeForce GTX 480 graphics card [18]. This is comparable to the expected performance of SWIPE on a quad-core CPU.

SWIPE scaled almost linearly with the number of threads used up to 12 threads, corresponding to the number of physical cores available. The X5650 processors features hyper-threading which makes it possible to obtain extra performance using more than one thread per physical core, unless all execution units of busy. Almost no increase in performance beyond 12 threads was observed, so apparently the execution units are fairly busy when SWIPE is running on 12 threads. SWIPE scaled much better than SWPS3 and STRIPED with multiple threads. The maximum speed of SWIPE was 6.5 times faster than SWPS3, the fastest of these, using the 375 residue query. When all programs were running on a single thread, SWIPE was about 2.5 times faster. There seems to be two equally important factors responsible for the differences in speed. Based on the single thread performance numbers, it seems like the use of the inter-sequence parallelisation approach instead of the striped approach is responsible for about half of the increase in speed. Efficient thread parallelisation seems responsible for the other half.

In the comparisons with the STRIPED software it should be noted that Farrar (2007) apparently reported the "scan time" and not the complete running time for the program, excluding the time needed to read the database sequences into memory. The full running time is reported here. For the other programs, it is difficult to separate the scan time from the rest of the time used. STRIPED reads the FASTA formatted files directly and not files formatted by NCBI's formatdb tool. It appears that STRIPED initially parses the entire FASTA file and reads the database into memory using non-threaded code, resulting in low performance when using several threads and measuring the complete running time. Excluding the time for database reading leads to shorter run times and higher performance numbers. On the other hand, it is probably faster to read files in the binary NCBI format than parsing the FASTA text format.

The performance of SWIPE was not very dependent on query length, except for rather short and very long queries. Overhead costs incurred for each database residue probably reduced the performance of SWIPE for the shortest queries. For the longest queries there was a performance decrease when many threads were running. This is probably due to the effects of memory caches, of which some are shared between cores. The programs based on Farrar's approach were considerably more dependent on query length and performed better with increasing query length. The reason for this is probably that as the width of the stripes increase, the relative importance of the dependency between the stripes is reduced.

SWIPE was about twice as fast as BLAST using the BLOSUM50 matrix, while BLAST was twice as fast as SWIPE using the BLOSUM62 matrix. BLAST performance was found to be very dependent on the scoring matrix. The reason may be that BLAST in its heuristics uses an initial hit score threshold (*T*) that has a fixed default value (11) independent of the score matrix specified. Score matrices with relatively high expected values, e.g. BLOSUM50, will then trigger more initial hits than other matrices, e.g. PAM30.

If one would like to search using a query profile (position-specific scoring matrix) instead of a query sequence, the computation of the temporary score profile need to be carried out for every position of the query, not just for the 20 possible amino acid residues. This has not been implemented, but the resulting reduction in speed has been estimated to 30%.

The software described here should be considered a prototype indicating the performance potential of the approach. A later version that at least computes the actual alignments (not just the alignment score) and the statistical significance of the matches is planned.

## Conclusions

Efficient parallelisation using SIMD on standard hardware now allows Smith-Waterman database searches to run considerably faster than before. In the new tool SWIPE, residues from sixteen different database sequences are compared in parallel to one query residue. Using a 375 residue query sequence a speed of 106 billion cell updates per second (GCUPS) was achieved on a dual Intel Xeon X5650 six-core processor system, which is more than six times faster than software based on Farrar's approach, the previous fastest implementation. Furthermore, for the first time, the speed of a Smith-Waterman based search has been shown to clearly exceed that of BLAST at least with one particular scoring matrix.

Since the slow speed has been the major drawback limiting the usefulness of Smith-Waterman based searches, the approach described here could significantly widen the potential application of such searches. Other applications that require optimal local alignment scores, like short read mapping [22] or genome sequence assembly [23] could also benefit from improved performance of this method. The approach used here may probably also be applied to HMM-based searches.

## Supplementary Material

Additional file 1 **Source code**. The source code of SWIPE version 1.0, as well as a binary executable for 64-bit Linux and score matrices are included in this gzipped tar archive file. Click here for file
